# Composition and Factors Affecting Quality of Bovine Colostrum: A Review

**DOI:** 10.3390/ani9121070

**Published:** 2019-12-02

**Authors:** Kamila Puppel, Marcin Gołębiewski, Grzegorz Grodkowski, Jan Slósarz, Małgorzata Kunowska-Slósarz, Paweł Solarczyk, Monika Łukasiewicz, Marek Balcerak, Tomasz Przysucha

**Affiliations:** Animal Breeding and Production Department, Warsaw University of Life Sciences, Ciszewskiego 8, 02876 Warsaw, Poland; marcin_golebiewski@sggw.pl (M.G.); grzegorz_grodkowski@sggw.pl (G.G.); jan_slosarz@sggw.pl (J.S.); malgorzata_kunowska_slosarz@sggw.pl (M.K.-S.); pawel_solarczyk@sggw.pl (P.S.); monika_lukasiewicz@sggw.pl (M.Ł.); marek_balcerak@sggw.pl (M.B.); tomasz_przysucha@sggw.pl (T.P.)

**Keywords:** calf, calving, quality, immunoglobulin, mammary gland, colostrum

## Abstract

**Simple Summary:**

In an attempt to improve the most important production traits of dairy cows, breeders omit the problem of calf rearing, whose regularity has a major impact on subsequent dairy and reproductive use. Therefore, it should be made clear to farmers that one of the ways to improve profitability is to improve the quality of colostrum. The most critical time for calves is the first 2 weeks, when the most falls occur, which may result from disorders of the digestive system and contribute to poor quality of colostrum or poor husbandry. Colostrum possesses a number of properties, such as nourishing, energetic, protective, but also purgative. It activates peristalsis, thus the excretion of meconium, therefore preventing its excessive densification and problems with excretion. Colostrum contains bioactive components with immune enhancing properties: Immunoglobulins, lactoferrin, lysozyme, lactoperoxidase, α-lactalbumin, β-lactoglobulin, or fat that carries important vitamins and polyunsaturated fatty acids. The concentration of the above-mentioned compounds is variable and depends on many factors, including breed, productivity, parity, feeding intensity, season of the year, and/or production system.

**Abstract:**

Colostrum as a secretion of the mammary gland is produced and accumulated in the final stage of pregnancy and in the first days after calving. It is designed to provide the calf with the necessary nutrients and biologically active ingredients. One of the most difficult periods in the life of animals is their rearing, and the most sensitive are the first days after birth. This is the time when most falls occur, and they are caused by mortality and morbidity, even at the level of 30%. Such losses affect the performance and profitability of animal production (the percentage of animals intended for reproduction or fattening is reduced and the intensity of selection in the herd is also reduced). Both diseases and mortality are the cause of serious economic, production, and breeding losses, which are the result of weak immune mechanisms. The adaptability of calves to the environment is determined by their immune status. Colostrum has a regulating function and stimulates the young organism to grow, and it has properties that support the functioning of systems: Endocrine and immunological. For colostrum to fulfil its role, it must be administered immediately after birth, because the immunoglobulins it contains are absorbed during the first 16–27 h after the birth of the calf, preferably within 2–4 h of age. Blood from calves that have been properly calved should have an antibody concentration of 15g/L (24–48 h of age). Therefore, immunoglobulins are the most important factor affecting infectious immunity; an adequate concentration of immunoglobulins in calves’ blood is related to their survival and health. It is the intent of this review to synthesize and summarize the information currently available on colostrum, as well as to discuss the interpretation of the results.

## 1. Introduction

Colostrum constitutes the secretion of mammary glands which is produced directly before giving birth and over the course of the following days [1,2]. It is immensely significant in terms of life during the perinatal period, because owing to it, calves gain immunity and cell-mediated immunity [3,4,5]. Colostrum’schemical composition differs in various types of animals, which is shown in Table 1. Colostrum constitutes a rich source of both immunoglobulins, as well growth factors such as insulin-like growth factors (IGF, I and II), platelet-derivedgrowth factor (PDGF), epidermal growth factor (EGF), transforming growth factor β-2 (TGF-β2), growth hormone (GH), and the cytokines interleukin 1-β (IL-1 β)—which serve as the stimulants and mediators in many processes taking place in cells [6,7]. It includes many antimicrobial, anti-viral, antifungal, and immunoregulating substances [8]. The most general definition of cow colostrum is that it is a thick, yellow, and slightly acidic (pH 6.4) liquid. Colostrum includes biologically active elements and bacteriostatic substances such as enzymes, hormones, polyamides, nucleic acid derivatives, amino acid [9]. The bacteriostatic substances include immunoglobulins, lactoperoxidase, lactenins, lactoferrins, lysozymes, and leukocytes. Among the above-mentioned elements, proteins are the ones most focused on, and especially immunoglobulins which constitute the main source of innate immunity concerning bacterial infections [1,3,4,10].

## 2. Colostrum’s Chemical Composition

Colostrum’s composition changes with each hour. Its biological and nurturing value decreases over time (Table 2). Time constitutes a critical element for feeding calves colostrum. That is why it is so important to provide colostrum immediately after birth (0.5–1 h). The ability to absorb immunoglobulins from colostrum decreases by 1/3 as soon as 6 h after birth, and by 2/3 after 12 h, and an intestinal barrier appears after 24 h [11,12,13,14].

Immunoglobulins are mono- or polymeric proteins, formed by two light and two heavy polypeptide chains which are connected by disulfide bonds into a Y-shaped particle. Immunoglobulins are divided into classes differing in their physicochemical, biological, and immunologic properties. The following Ig classes have been determined for humans and animals: H, G, M, A, D, E, and they occur in various secretions (blood, milk, colostrum, tears, and mucus) [15]. Immunoglobulins present in cattle include IgG, M, and A, where G immunoglobulins constitute 65–90%. There are two isotypes of Ig: IgG1 and IgG2. These Ig work together to provide the calf with passive immunity (immunity provided by the cow and not synthesized by the calf) until the calf’s own active immunity develops [16].The ratio between G1 and G2 in colostrum is 35:1. Plasma cells of the lymphatic system are responsible for producing G immunoglobulins. The M immunoglobulins constitute only 8–10% of colostrum’s Ig protein; in newborns this fraction constitutes 20–30% in reference to adults, and the absorption from intestine during the first hours of life is 95%. Ig M constitutes a factor which conditions antibody-mediated antiseptic immunity [15]. In cattle, A class immunoglobulins constitute 7–10% of the colostrum’s immunologic potential. Owing to the calf high absorption activity, antibody content within the calf blood serum rapidly increases and is also kept at a relatively high level until the 2nd–3rd week of life [10]. Colostrum is produced in the mammary gland primarily during the last three weeks of pregnancy. During that period, IgG1 and IgG2 immunoglobulins are transferred to the mammary gland. The IgG1 immunoglobulins are transferred passively, whereas IgG2 immunoglobulins are transferred selectively, achieving a much greater concentration in the colostrum than in serum. The entire process of transferring immunoglobulins becomes faster during the final stage of pregnancy, when the udder involution also takes place within the newly created gland epithelium cells. The highest concentration of IgG1 in colostrum appears a few days before calving, and then it becomes less, mainly due to an increasing amount of colostrum in the mammary gland [17]. Calves are born essentially agammaglobulinemic [13], and at the time of parturition, maternal Ig from the circulating pool in the blood is actively concentrated in the secretion of the mammary gland [2]. Passive transfer of immunity is essential for the short-and long-term health of dairy calves [18]. Absorption of Igfrom colostrum is time dependent, and delays in reaching the small intestine could negatively affect both the rate and amount of absorption [19]. Immunoglobulin ingested by the calf is taken up by the epithelial cells of the small intestine and passes into the lymph spaces and then into the blood circulation through the thoracic duct. This transfer mechanism (passive transfer) starts to decline approximately 12–23 h after birth and ceases, on average, at 24 h [20]. Additionally, economic losses associated with failure of passive transfer have been estimated to average $65 per calf when accounting for calf mortality, morbidity, and decrease in average daily weight gain [21].

Calves require fat and protein for energy and muscle development, as well as growth factors and many other nutrients that are concentrated in colostrum [22]. The newborn calfhas relatively few energy reserves, with lipids comprising only 3% of body weight [23]. The fat content of colostrum is greater than that of milk [10,24].

There is also a different group of proteins which are very significant in terms of the colostrum’s bacteriostatic and germicidal properties, and which consist in non-specific antibiotic agents: lysozyme, lactoferrin, and lactoperoxidase [10].

Lysozyme is germicidal concerning almost all body fluids. The only resistance has been determined in the case of lactic and propionic acid bacteria. Furthermore, its activity increases at the presence of immunoglobulins [25,26,27]. Thanks to its immunity against digestive proteases, it may remain active while going through the smaller intestine. The concentration of lysozyme and immunoglobulins of the IgG and IgM classes in the colostrum shows the opposite trend in the first and second milking after calving [25]. The increase in the level of lysozyme is associated with a decrease in the concentration of these Ig.

Lactoferrin (Lf) concentration of colostrum is between 0.34 and 1.96 g/L [1,10,28,29]. Thanks to binding iron, it has a bacteriostatic effect and halts the development of bacteria [30,31]. However, it does not have a bacteriostatic effect on lactic acid bacteria and *Streptococcus* requiring small amounts of iron. It loses its activity in an acidic environment, and its bacteriostatic properties may change in the intestine. Robblee et al. [32] proved that lactoferrin may be a beneficial dietary supplement for newborn calves, because it improves recovery rates. In addition, Habing et al. [33] showed that Lf significantly reduces mortality. Lactoperoxidase also possesses bacteriostatic and germicidal properties [34]. 

Lactose is the most representative carbohydrate, and is primarily a source of energy for the calf [35]. It consists of two hexoses: Glucose and galactose. Lactose plays an important role in the correct development of new-born mammals as it is an important source of energy, indispensable for such organs as the heart, liver, and kidneys. Additionally, it stimulates the functions of the spinal cord’s and the brain’s nerve cells [36]. 

Kowalski [37] showed the amount of basic food products in the colostrum (energy, protein) is constant, but the amount of mineralsis a result of meeting the cow’s needs concerning various elements during the perinatal period. The author points especially to selenium and iodine;those deficiency may result in a poor growth, muscular dystrophies, and a tendency for diarrhea. Deficiencies of all elements, as well as micro- and macro-elements, have a negative impact on the upbringing of calves. According to Berleć and Traczykowski [38], in extreme cases, they lead to severe diseases or even cachexia and death. Colostrum includes from 2 to 10 times more minerals (except for potassium) than milk. Theutilization rate of these minerals is at 92–98% [5]. Their high concentration results in the colostrum’s bitter taste [39]. The amount of minerals between colostrum and milk also changes, which is presented in Table 3. Tsioulpas et al. [40] reported that the concentrations of calcium and phosphorus decreased from 54.2±6.7 and 52.8±8.6 mM, respectively, at parturition, to 33.5±4.1 and 30.0±4.1 mM, respectively, after 15 days. On the other hand, Kehoe et al. [1] reported mean concentrations of calcium and phosphorus in colostrum, which were approximately 4- and 5-fold greater than the concentrations found in milk. Abd El-Fattah et al. [24] reported a decrease in the levels of copper, iron, and zinc in colostrum over the first 336 h post-partum. 

Vitamins included in colostrum are both fat-soluble (A, D, E, and K) and water-soluble (thiamine, riboflavin, pyridoxine, cobalamin, niacin, biotin, pantothenic acid, folic acid, nicotinic acid, and choline) [7]. Marnila and Korohnen [41] reported that the concentrations of thiamine, riboflavin, folate, pyridoxine, and cobalamin are greater in colostrum than in milk, while the levels of pantothenic acid and biotin are lesser in colostrum and the concentration of vitamins A and E are generally greater than in milk [42]. Together with magnesium and zinc compounds, vitamins play a very important role in supporting the defense mechanisms of calves and creating theirown infection immunity. Vitamins E and C possess antioxidizing properties and help to stabilize membranes (macrophage, granulocytes, and lymphocytes). A significant role is played by vitamins A, D, and E, and their deficiency directly translates into lessening the immune-suppressing capabilities of the body [39]. The processes of digestion and absorption of lipids are closely related and proceed according to the following scheme: Emulsification, enzymatic hydrolysis, formation of micelles, and proper absorption [43]. However, transfer of vitamin E into colostrum does not appear to occur via a passive mechanism associated with the transfer of lipids [44], but rather by a mechanism involving low-density lipoproteins [45]. Kehoe et al. [1] reported that the concentration of vitamin E in colostrum ranges from 60 to 1040 µg/100 g. Vitamin E participates in the first line of defense against reactive oxygen species (ROS), effectively suppressing singlet oxygen. In the second line of defense, it reacts with free peroxide radicals of the lipids, inactivating them, interrupting their production, and inhibiting the sequence of free-radical chain reactions damaging cells. However, to be effective, the co-antioxidant vitamin C is required. The antioxidant effect of vitamin E increases the stabilization of cell membranes and other substances sensitive to oxidation. It prevents the oxidation of polyunsaturated fatty acids, helping to maintain the elasticity and tightness of blood vessels [46]. The amount of vitamin A changes the most within colostrum during the days after calving [24]. Vitamin A regulates the speed of the gene transcription process by linking to the appropriate nuclear receptors. Vitamin A is also necessary in the synthesis of mucopolysaccharides co-creating connective tissue and glycoproteins, which are a constituent element of body fluids and membrane proteins. Vitamin A helps stabilize and regulate the permeability of protein–lipid elements of cellular and subcellular membranes [46]. A deficiency of retinol has an impact on lowering the protective capability of the epithelium, and increases the chance for diarrhea caused by *Escherichia coli*. In the case when a cow receives an insufficient amount of β-carotene, the colostrum may prove to be lacking a proper amount of this element. Such cases happen during winter and early-spring calvings, due to the feeding of cows preserved feeds, often not containing a proper amount of β-carotene [39]. The prophylactic property of vitamin A is one of the most efficient when we provide it to the calf in the form of a water suspension 1–2 h after birth. This has an impact on creating the immunity of the intestine epithelium tissue against *Escherichia coli*. Additionally, the amount of β-carotene consumed is related to the general functions of this vitamin—a catalyst of many metabolic functions (protein and energetic), as well as the nervous system and endocrine glands [47]. 

## 3. Colostrum Quality Evaluation

It is advised to provide colostrum often—3 feedings/day to achieve a minimum of 6 L/day, during the first two days of the calf’s life [48]. Additionally, Osaka et al. [49] concluded that failure of transfer of passive immunity in newborn calves may be avoided if calves consume ≥3 L of colostrum with Ig concentration >40 mg/mL within 6 h after birth. A quick milking of colostrum and providing it to the newborn is also important due to the changes undergoing in its composition during the following hours (Table 4). Current industry practices include feeding colostrum through an esophageal tube or a nipple bottle. Desjardins-Morrissette et al. [19] reported that tube-fed calf consumed 0.5 ± 0.13 L more colostrum in their first meal than bottle fed calf. On the other hand, Godden et al. [50] found that feeding colostrum with a nipple bottle increased serum concentrations of Ig when calves were fed 1.5 L of colostrum, compared with an esophageal tube.

The level of Ig in colostrum may be evaluated using various methods. The radial immunodiffusion method or acolostrometer analysis are regarded as the standard methods. The easiest method to determine the quality of colostrum is measuring its density, becauseits density is correlated with the Ig content. A device used for this purpose is the colostrometer. According to Horecka [51], it is one of the basic and most simple devices used to assess the quality of colostrum. The colostrometer measures the density of the liquid, which makes it possible to read the content of immunity proteins, meaning immunoglobulins, assigned to a specific density. The colostrometer‘s scale includes various colors, indicating specific quality levels of the colostrum: Green is usually used for good quality, yellow for average, and red for bad quality colostrum. If the colostrometer presents the color green, then the calf should receive the colostrum in an amount constituting 10% of its body mass. Such a color indicates that the colostrum includes more than 50 mg/mLof antibodies. 

According to experts, in comparison to a colostrometer, a refractometer is a more precise device. It allows to assess the concentration of aqueous solutions basing on the refractive index. The light refraction index processed through the Brix scale with the use of optical and electronic devices has been listed as a valuable indicator differentiating good quality colostrum from poor quality colostrum [52]. When it comes to using a colostrometer, a large sample is needed, but it constitutes an indirect measurement of the Ig content. Unfortunately, the correlation between specific gravity and the Ig content is very low (R^2^ = 0.38) [53]. Most refractometers include a system of automatic temperature compensation (ATC), which allows to avoid the need of using data from calculation tables to correct the measurement. A refractometer is much easier to use and more sensitive. Its advantages are that a small amount of the analyzed materials is needed, only a few drops of colostrum, and a high correlation with the Ig concentration (R^2^ = 0.6–0.7). Additionally, a refractometer allows to measure the concentration of immunoglobulins in the blood’s serum [52]. 

In the case of a radial immunodiffusion (RID), a specialist laboratory is necessary, and its disadvantages are both the time it takes (period between taking the sample and results) and the availability of the service [54]. 

## 4. Discussion

### Factors Shaping the Quality and Chemical Composition of Colostrum

Calves up to 6 h after birth should receive the first portion of colostrum, with an average content of 100–200 g of immunoglobulin if colostrum is classified asgood to very good quality [49,55]. Delay in consumption significantly increases the calf’s risk of disease and mortality [56]. This requirement is satisfied only ifcolostrum is of good tovery good quality—which is becomingincreasingly rare in the high performance dairy herds [10]. As reported by Ganchev et al. [57], the concentration of Ig in the serum of calves after drinking colostrum should be at least 10 g/L (to prevent failure of passive transfer), with a concentration of 5–10 g/L, a partial deficiency can be found. Kryzer et al. [58] reported that calves fed heat-treated colostrum (heat-treated in a Perfect Udder bag at 60 °C for 60 min and then stored at −20 °C, or heat-treated in a batch pasteurizer at 60 °C for 60 min and then stored at−20 °C in a Perfect Udder bag) experienced significantly improved apparent efficiency of absorption of Ig at 24 h and serum Ig concentrations. Osaka et al. [49] reported that serum Ig concentration in calves at 24 h was significantly influenced by the mass of Ig consumed. Additionally, Furman-Fratczak et al. [59] showed that the morbidity and intensity of disease course were lowest in heifer calves with serum Ig concentration exceeding 10 g/L at 30–60 h of life. 

To achieve successful passive transfer of immunity, it has been suggested that a calf needs to receive at least 150–200 g of Ig within 2 h of birth [60]. This can normally be achieved by feeding 3–4 L of high-quality colostrum with Ig >50 mg/mL [61]. When colostrum quality is poor or unavailable, colostrum replacers (CRs) may be an alternative. Lago et al. [62] reported that CR-fed calves had a lesser probability of receiving contaminated liquid feed and performed similar in terms of health compared with calves receiving high-quality colostrum. Therefore, CRs can be an alternative to poor-quality colostrum. Additionally, Pithua et al. [63] reported that the risk of *Mycobacterium avium* ssp. *Paratuberculosis* infections werereduced by almost 50% when using a plasma-derived CR compared with using colostrum.

Colostrum differs significantly from milk in terms of physiochemical properties (acidity, color), but also concerning the proportions of the main ingredients, both the protein and fatty fractions (richer with minerals and proteins, but containing less carbohydratesand lipids) [8]. Similarly to milk, colostrum contains more than 250 various active chemical compounds (Table 5). In comparison to milk, it includes an increased share of elements synthesized from blood: Proteins, including immunoglobulins, minerals, vitamins, and less lactose [64]. Some of specific amino acids in colostrum are almost always greater than in milk, except for glutamic acid, proline, and methionine (Table 6). According to Lach [65], in comparison to milk, colostrum contains 2 times more dry matter, 3 times more minerals, 5 times more proteins, and also more fat-soluble vitamins. Christiansen et al. [8] stated that the differences in the basic composition of bovine colostrum and mature milk begin to allow insight into the differences in the biological functions of the two materials.

The quality of colostrum varies, and that variability is determined by both individuals and environmental factors: Parity, pre-partum diet, season, breed, dry-period length, vaccination of the dam, and delayed colostrum collection [7,10,66,67,68]. Other factors, such as abortions [69], and the health status of the cow [70,71] have also been found to influence colostrum quality.

According to Szulc and Zachwieja [5], the herd factor has the biggest effect on the composition of cow colostrum (excluding α-lactalbumin and β-lactoglobulin). Which means that the intensity of feeding and the conditions in which the cows are kept before calving are most important when it comes to the quality of produced colostrum [1,10]. Other factors which have an impact on the variability of analyzed characteristics include age of the cows and calving season. The colostrum’s efficacy is negatively correlated with its density and also its contents, which means that as the amount of produced colostrum increases, its quality worsens. Silva-del-Río et al. [68] reported that when colostrum yield increased from low (<3 kg), medium (3–6 kg), to high (>6 kg), Ig concentration decreased. However, the mass of Ig transferred into colostrum does not appear to be related to the size of the mammary gland [72].

Colostrum’s composition is characterized by a genetic diversity, which concerns most of the analyzed factors. The heritability of the immunoglobulin concentration level in the colostrum of Holstein–Friesian cows reported by Szulc and Zachwieja [5] is h^2^ = 0.50. Lesser levelshavebeen reported by Gilbert et al. [73] in the colostrum of US Hereford, Angus, and Simmental cows (h^2^ = 0.41), and by Conneely et al. [74] in Holstein–Friesian, Jersey, Jersey x Holstein–Friesian crossbreeds, Norwegian Red, Norwegian Red x Holstein–Friesian crossbreeds. 

An internal factor which has a direct impact on the achieved daily weight gain during the period of feeding calf colostrum is the paternal effect. According to Kuczaj et al. [75], groups of offspring from specific bulls differed when it came to phenotype values such as body weight in the 5th day, and the daily weight gain between the 1st and 2nd day. 

Breed can affect colostrum quality, and traditionally Holsteins have been thought to have lesser colostral Ig concentrations than other dairy breeds. According to Nardone et al. [76], the ratio of protein, fat, lactose, and dry matter in the colostrum of Holstein–Friesian cows is, respectively, 16.6%, 6%, 3.2%, and 25.8%. Whereas, for Holstein–Friesian and Simental cross-breed cows, there is an increase of dry matter, fat, and lactose [77]. According to Wroński and Sosnowska [78], a greater level of dry matter and a lesser level of lactose are formed in the colostrum of Black–White cows in comparison to Angus cows. According to Maunsell et al. [70], colostrum from Ayshire and Brown Swiss breed cows is characterized by a lesser density (respectively: 1.0488 g/dm^3^ and 1.0473 g/dm^3^) in comparison to the colostrum of Holstein–Friesian cows (1.0524 g/dm^3^). It has been determined that a greater content of Ca, Mg, andP is present in the colostrum of cows with a β-lactoglobulin BB genotype, in reference to the colostrum of cows with other genotypes [79].

The dry period may have a significant impact on both the quantity and quality of produced colostrum. Especially, milking cows until the very calving or premature milking due to swelling before birth. It is estimated that the dry period should last 5 weeks, during which secreting epithelium cells regenerate and the process of lactogenesis takes place. During the dry period, the precolostrum undergoes changes. There is a dominance of IgA in the precolostrum, while colostrum is richer in Ig. Milking cows prior to calving has an impact on lowering the level of immunoglobulins in the colostrum, leading to a lesser capability to pass the passive immunity to newborns. The highest levels of fat and protein in the colostrum were determined in cows that had a dry period that lasted 4 weeks, while the highest level of lactose was determined for the cows that had a 2-week-long dry period before parturition [80]. 

The necessity to perform pre-calving milking leads to extruding immunoglobulins collected in the udder during the final stages of the pregnancy. The secretion of the mammary gland in cows milked before calving shows a lesser content of protein, fat, and dry matter, a slightly greater amount of lactose, and a lesser amount of whey proteins (at more than 40%), immunoglobulins (at circa 30%), β-lactoglobulin (20%), and α-lactoalbumin (90%) [5]. Whereas, the duration of the pregnancy does not have a significant impact on the composition of cow colostrum. 

The age of the cow also constitutes a factor conditioning the properties of colostrum [23,81,82]. Significant composition changes may occur for cows during following lactations. Multiparous cows produce more colostrum with a greater concentration of dry matter, total proteins, and whey proteins, including immunoglobulins [5,7,23,83,84]. The highest immunoglobulin levels can be found in the colostrum of cows thatare in the 3rd–5th lactation, and the lowest in the primiparous cows. Therefore, it can be stated thatcolostrum production is usually lesser in first lactation, suggesting weaker mammary development and potentially reduced transport immunoglobulins into the mammary gland [85]. Additionally, Donovan et al. [81] reported that the increase in Ig concentration is due to an increase in antigenic exposure and incidence of disease. 

The impact of cow or mammary gland diseases on the quality of colostrum has not been unequivocally defined, and research results are discrepant. However, the prominent opinion is that udder diseases, chronic acidosis, and ketosis have an impact on lowering the level of immunoglobulins and quality of the produced colostrum. Cows with a mammary gland inflammation produce colostrum with a smaller ratio of trypsin inhibitor, leading to limiting the assimilability of Ig for calf. Research by Błaszczykowska and Twardoń [86] and Ontsouka et al. [77] stated that udder inflammations have an negative impact on the colostrum’s value, and another negative impact is also reported on the level of assessment of colostrum immunoglobulins by calves.

According to Szulc and Zachwieja [5], difficult births may have an impact on lowering the quality and quantity of colostrum, and also on lowering passive immunity in calves. Also cesarean-section births may impact the amount and quality of the mammary gland secretion, or even stop its production. Also, the degree of the birth’s difficulty has a significant meaning in terms of the achieved daily weight gains during colostrum feeding periods, although it does not significantly differentiate the body weight of calf in the 1st and 5th days of their lives [75].

Poor sanitary condition of the cowshed reduces the quantity and composition of the colostrum. A greater Ig concentration has been reported for colostrum of cows kept in bedding-free cowsheds. However, according to research conducted by Szulc and Zachwieja [5], no differences have been determined concerning the level of proteins in colostrum of free stall cowsheds and those including bedding. 

After milking, the colostrum may be stored in open vessels at the temperature of 2–4 °C for 2–3 days. Storing colostrum in warmer conditions significantly alters both total bacteria count and pH [87]. Cummins et al. [88] reported that colostrum stored in warmer conditions had >42 times more bacteria present and resulted in 2-fold lesser serum Ig compared with colostrum that was pasteurized, untreated, or stored at 4 °C for 2days.

The storing period may be increased by taking advantage of chemical additives—acetic, formic, propionic, benzoic, and lactic acids, and the following cultures of bacteria for fermentation—*Streptococcus lactis, Streptococcus termophilus, Laktobacillus acidofilus, Lactobacilus bulgaricus*. Colostrum preserved in such a manner may be stored atroom temperature even for a few days. However, acidifying colostrum with bacteria is less advantageous due to the decomposition of lactose, which reduces its digestibility. It also has to be mentioned that the absorbability of all elements of an acidified colostrum is lesser than in the case of a fresh one. 

Heat treatment of milk and dairy products is aimed mainly at killing microorganisms and inactivating enzymes [7]. Godden et al. [51] reported that heating of colostrum to 60 °C for 120 min was sufficient to reduce the level of viable *Mycoplasma bovis*, *Listeria monocytogenes*, *Escherichia coli* O157:H7, *Salmonella enteritidis,* and *Mycobacterium avium* subspecies paratuberculosis below detectable limits. However, Saldana et al. [18] reported that heating colostrum reduced Ig concentration compared with the control by 9% when heated for 30 min and by 12% when heated for 60 min. 

A method for a long-lasting preservation of colostrum is freezing it at a temperature of −18 to −20 °C. It is advised to freeze it in water at atemperature lower than 60 °C, because immunoglobulins are very sensitive to greater temperatures. Freezing stops the multiplication of bacteria and microorganisms, but does not eliminate them, which may result in infecting the newborns fed with it. Stored frozen colostrum may last up to half a year. If during that period it is notnecessary for newborns, then it may be used to feed 2–3 week old calves as an addition to milk or a milk-substitute. This results in an intestinal colonization, and thus reducesthe risk of infection. It is a very important period because the colostrum immunity drops, and own immunity is low. 

The fact that it is impossible to preserve larger quantities of colostrum by freezing it acts in favor of taking advantage of a different method, namely, the dehydration process. Taking advantage of spray drying or lyophilization, both of these methods allow to protect elements sensitive to high temperatures against degradation. However, a negative impact on colostral fat has been observed with lyophilization. A large number of milk fat globules is destroyed, which makes the fat rancid quicker. That is why it is advised to use this method with fat-free colostrum. Lyophilization takes advantage of the sublimation method (drying in a lower temperature and vacuum). The Ig assimilability from dried and lyophilized colostrum is lower at circa 30% in comparison to fresh colostrum [5]. However, better results are achieved by adding dried or lyophilized colostrum to the mother colostrum. Drying colostrum allows for long storage periods and to manufacture colostrum agents complemented with the missing elements. It has been observed that it is possible to increase the assimilability of colostrum by adding various bio-agents to dried or lyophilized colostrum. An important criterion allowing to preserve colostrum is its weight and immunoglobulin content, which should be, respectively, 1.050 g/cm^3^ and 60 g/L. When it comes to the drying method, proper conditions should be kept so that the temperature of colostrum particles does not exceed 50–55 °C, which allows to maintain its biological value. Dried or lyophilized colostrum should be given to the calf after dissolving in water at the temperature of 40–50 °C. Dissolving it in fat-free milk has a negative impact on the assimilability of immunoglobulins (assimilability lesser at 19–36%) [5]. 

As stated by Demkowicz [64], there are other ways of preserving colostrum, such as pasteurization, UV irradiation, membrane filtration, or taking advantage of a pulsating electric field (PEF) and a concentrated microwave field (CMF). However, each of these methods results in a number of changes in the chemical composition of the colostrum. However, according to Lach [65], a colostrum-like agent constitutes only an emergency solution and cannot replace actual colostrum.Additionally, Denholm et al. [89] reported that use of potassium sorbate to preserve colostrum resulted in a lesser increase in total bacterial counts than colostrum stored without preservative or with yoghurt added. 

Research conducted over the course of 20 years shows that colostrum may be preserved and used in other ways apart from feeding calves. Various preservation methods provide the possibility to use it for prophylactic purposes with both humans and animals. The following products constitute examples:

- A sheep colostrum product, including 11.3% conjugated linoleic acid dienes (CLA); it is more efficient in suppressing the development of cancer cells than a commercial 80% Bio-CLA.
- Colostrinin, a protein complex from cow or sheep colostrum; it slows down Alzheimer’s disease.

Gastrogard-R^®^ is prepared from the colostrum of hyperimmunized cows and contains high antibody titers against four human rotavirus serotypes, as measured in a virus neutralization test. Knipping et al. [90] reported that preventing rhesus rotavirus induced diarrhea by Gastrogard-R^®^ early in life showed a diminished protection against epizootic diarrhea of infant micere-infection. 

Additionally, Huppertz et al. [91] reported that bovine colostrum is well tolerated and diminishes frequency of loose stools in children with *E. coli*-associated diarrhea.

## 5. Conclusions

The quality of colostrum varies, and that variability is determined by both individualsand environmental factors, such as parity, pre-partum diet, season, breed, dry-period length, vaccination of the dam, delayed colostrum collection, abortions, or health status of the cow. As is known, colostrum has a decisive meaning when it comes to the health of calves and results achieved in the future. This is why it is worth paying attention so that a good quality colostrum should be provided to calves.

## Figures and Tables

**Table 1:** Changes occurring in the chemical composition of colostrum from the first collection depending on the species of farm animals [5].

Species	Chemical Composition (g/kg)		
	Fat	Protein	Lactose
Horse	7	191	46
Cattle	36	130	31
Sheep	124	130	34
Goat	90	80	25
Pig	72	180	24
Dog	78	138	27
Rabbit	47	135	16

**Table 2:** Chemical composition of colostrum and milk (%) in particular hours after calving [11].

Specification	Protein	Casein	Albumin, Globulin	Fat	Lactose
Colostrum (h)					
0	16.8	4.1	12.7	6.7	2.9
6	11.7	3.5	8.0	6.1	3.5
12	6.3	3.1	3.2	4.4	3.9
24	5.5	2.9	2.6	4.1	4.1
48	4.8	2.8	2.0	3.9	4.2
120	3.6	2.7	0.9	0.8	4.5
**Milk**	3.2	2.6	0.6	3.8	4.6

**Table 3:** The content of minerals (%) in colostrum and milk [39].

Testing Time	Calcium	Magnesium	Potassium	Sodium Chloride	Phosphorus	Chloride
**At the time of calving**	0.256	0.037	0.137	0.074	0.235	0.118
**After 11 days**	0.130	0.011	0.153	0.036	0.113	-

**Table 4:** Changes in the composition of colostrum and milk of Holstein Friesians cows (%) [51].

Time from Calving	Water	Casein	Albumin, Globulin	Fat	Lactose
directly	66.4	5.57	16.92	6.5	2.13
after 12 h	79.1	4.47	8.98	2.5	3.51
after 24 h	84.4	4.23	2.63	3.6	4.24
after 48 h	86.3	3.91	1.23	3.7	4.51
after 6 days	87.9	2.76	0.75	3.7	4.78
after 25 days	87.6	3.0	0.5	3.8	4.6

**Table 5:** Basic chemical composition of colostrum and milk (g/100 mL) [5].

Component	Colostrum	Milk
**Protein** **including whey protein**	14.56 10.87	3.3 0.6
**Fat**	5.35	4.0
**Lactose**	2.03	4.8
**Minerals**	1.2	0.65

**Table 6:** The amino acid composition of colostrum and milk (g/kg) [5].

Amino Acids	Colostrum	Milk
Aspartic Acid	42.95	28.83
Threonine	33.26	14.64
Serine	44.95	19.74
Glutamic Acid	88.84	91.12
Proline	25.96	56.98
Cystine	8.51	2.39
Glycine	15.65	5.96
Alanine	15.79	11.42
Valine	28.33	16.95
Methionine	9.31	12.00
Isoleucine	15.1	13.67
Leucine	47.30	35.94
Tyrosine	39.56	15.34
Phenylalanine	25.22	17.16
Histidine	14.60	12.12
Lysine	40.90	28.51
Arginine	14.40	10.22
